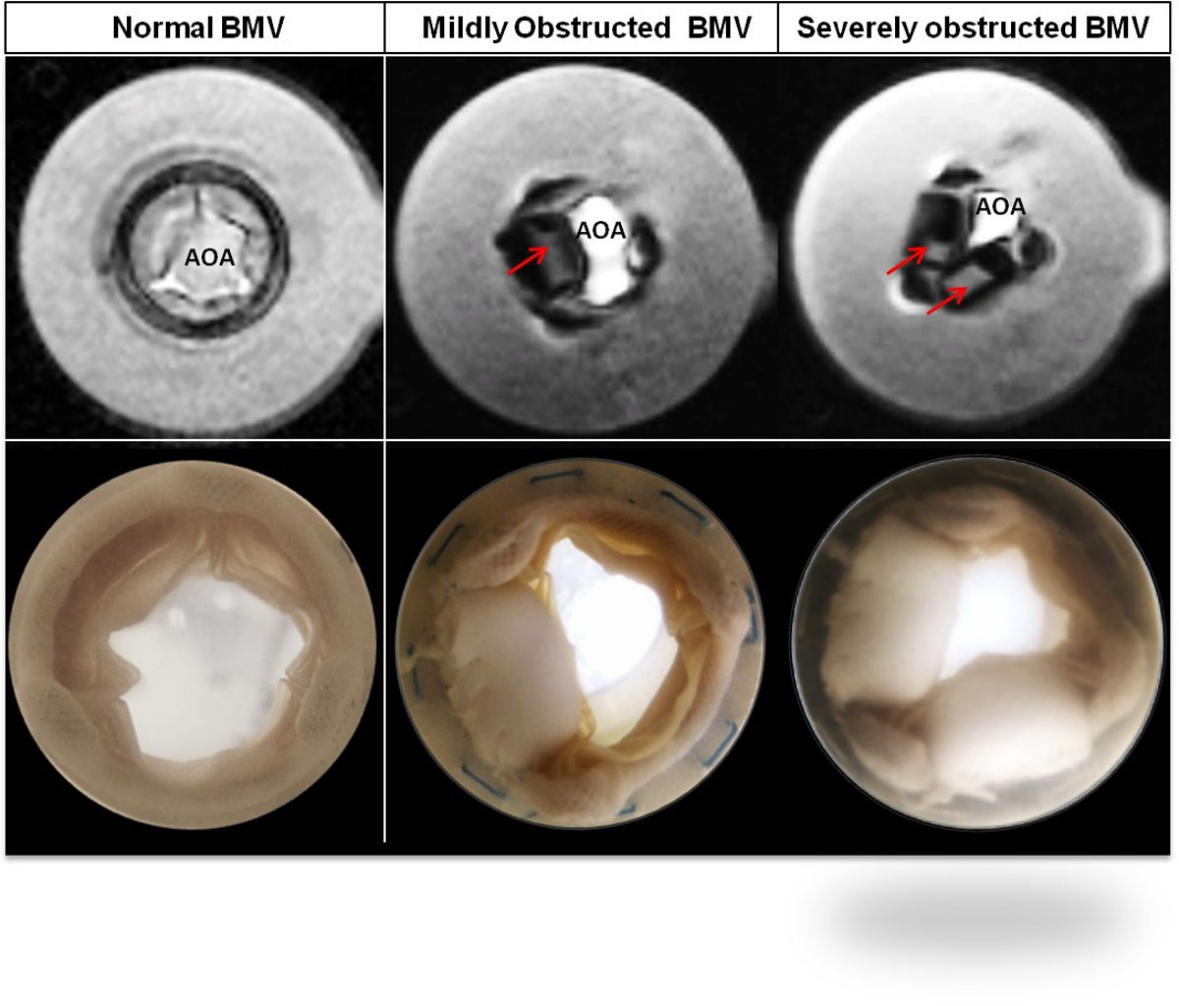# Cardiac MRI functional assessment of bioprosthetic mitral valves: an in vitro comparison against doppler echocardiography

**DOI:** 10.1186/1532-429X-17-S1-P335

**Published:** 2015-02-03

**Authors:** Dimitrios Maragiannis, Matthew Jackson, Karen Chin, Stephen Igo, Kyle Autry, William A Zoghbi, Dipan J Shah, Stephen H Little

**Affiliations:** 1 Cardiology, Houston Methodist Hospital, Houston, TX, USA

## Background

Evaluation of bioprosthetic mitral valve (BMV) function remains an important goal in clinical practice. We hypothesized that cardiac MRI (CMR) is accurate in measuring transvalvular flow volume and anatomic orifice area (AOA) in normal and obstructed BMVs in an *in vitro* model.

## Methods

Four sizes of normal BMVs (27, 29, 31, 33mm) and 4 stenotic BMVs (27mm and 29mm; with one or two leaflet obstructions representing mild and severe stenosis) were evaluated in a pulsatile *in vitro* heart chamber under 3 flow conditions (70ml, 90ml, 110ml/beat) at rate of 70 beats/min. All constructs were evaluated by phase-contrast (PC) CMR; the reference standards for comparison were continuous wave Doppler imaging, and standard flowmetry.

## Results

CMR methods accurately measured transvalvular flow and correlated strongly with flow transducer measurements (N=24; r=0.961, p<0.001). Bland-Altman analysis demonstrated a 95% confidence interval from -9.5 to 8.6 ml/beat across the range of both normal (mean EOA=1.82 cm^2^) and obstructed valves (mean EOA=0.96 cm^2^). Anatomic orifice area (AOA) correlated well with Doppler effective orifice area (EOA) (r=0.986, p<0.001); AOA was larger than EOA as expected (mean difference was 0.68 cm^2^). Furthermore, peak velocity by phase contrast CMR correlated strongly with Doppler peak velocity (r=0.984, p<0.001). Bland-Altman analysis revealed a 95% confidence interval of -44.4 to 19.36 cm/sec.

## Conclusions

PC-CMR methods accurately assess bioprosthetic transvalvular flow, peak velocity and orifice area. The CMR methods described hold promise in evaluating bioprosthetic mitral valve function in cardiac MRI studies, particularly when Doppler methods are unobtainable or discordant.

## Funding

American Heart Association grant #11BGIA5840008.

**Figure 1:**